# Schema modes in eating disorders compared to a community sample

**DOI:** 10.1186/s40337-015-0082-y

**Published:** 2015-11-19

**Authors:** Daniel Talbot, Evelyn Smith, Alethea Tomkins, Robert Brockman, Susan Simpson

**Affiliations:** Clinical and Health Psychology Research Initiative (CaHPRI), School of Social Sciences and Psychology, Western Sydney University, Locked Bag 1797, Penrith South DC, NSW 2751 Australia; School of Psychology, Social Work and Social Policy, University of South Australia, Adelaide, NSW Australia; Graduate School of Health, University of Technology Sydney, Sydney, NSW Australia

**Keywords:** Schema mode therapy, Eating disorders, Schema mode inventory, Eating disorder examination questionnaire, Female, Australia

## Abstract

**Background:**

The aim of this study was to examine the association between eating disorders (ED) and schema modes, and identify which specific schema modes are associated with particular eating disorders, including anorexia nervosa (AN), bulimia nervosa (BN) and other specified feeding or eating disorder (OSFED).

**Methods:**

A total of forty seven women with eating disorders and 89 women from the community participated in this study. Eating disorder diagnosis was determined by a clinician treating the eating disorder and was confirmed on the basis of Body Mass Index (BMI) and the Eating Disorder Examination Questionnaire (EDE-Q). The Schema Mode Inventory (SMI) was used to explore the association between schema modes and eating disorder diagnostic status.

**Results:**

A series *t*-tests revealed that when compared to the community sample, the ED group scored significantly higher on 10 out of 12 maladaptive schema modes, and significantly lower on both adaptive schema modes. A series of planned contrasts revealed that the AN, BN, and OSFED groups each scored significantly higher than the community sample group in the majority of maladaptive schema modes, with slight variations between groups. Further, AN, BN, and OSFED groups each scored significantly lower than the community sample group for the two SMI scores categorized as adaptive. All Cohen’s d that reached significance ranged 0.55-2.24.

**Conclusions:**

The current study shows a tendency for females with eating disorders to rely on maladaptive schema modes more frequently, and more adaptive schema modes less frequently compared to a community sample. These findings provide initial empirical support for a schema mode model of eating disorders.

## Schema modes in eating disorders compared to a community sample

Currently, the treatment of choice for adults with eating disorders (EDs) is cognitive behavioural therapy (CBT). However, many individuals with EDs do not benefit from CBT [1–8] and there is thus an urgency to investigate new treatment models. Because 69 % of ED sufferers may meet diagnostic criteria for a personality disorder, there is a need for a treatment model that specifically speaks to the role of early experiences in the development of “core” schema-level beliefs, as well as the coping mechanisms that maintain these underlying structures. Schema therapy is one such treatment model that addresses rigid schema beliefs [9]. Schema theory asserts that every individual has universal core emotional needs. If these needs are not adequately met, it can result in long-standing patterns of maladaptive thinking, feeling, behaving and coping [9]. These themes or patterns of thinking effectively act as crucial maintenance factors in ED.

Schema-mode therapy seeks to address these universal core emotional needs by strengthening adaptive schema modes and weakening maladaptive schema modes, [9] and has been found to be an effective treatment for a variety of mental health and personality difficulties [10, 11]. Schema modes can be clustered into four categories: (1) innate child modes that can become maladaptive as a result of significant unmet core childhood needs, (2) maladaptive coping modes, (3) maladaptive (internalised) parent modes, and (4) adaptive modes.

Evidence suggests that maladaptive schemas are more strongly held by individuals with anorexia nervosa (AN) and bulimia nervosa (BN) compared to healthy controls [12], and there is some evidence of schema-mode therapy being effective for EDs [13]. One study suggests that individuals with EDs display a greater prevalence of maladaptive schema modes compared to individuals without EDs, and different pattern of modes compared to obsessive compulsive disorder [14].

The current study aimed to provide further evidence for the association between schema modes and EDs. It was hypothesised, in line with previous findings [14], that individuals with EDs would score significantly higher on maladaptive schema modes, and lower on adaptive schema modes, compared to a community sample, as measured by the Schema Mode Inventory (SMI). This study also sought to explore the differences in schema modes amongst individuals with AN, BN, and other specified feeding or eating disorder (OSFED) compared to a community sample.

## Method

### Participants

Forty seven female participants with EDs were recruited via ED specialists. Diagnoses were based on clinical definitions of AN, BN, binge eating disorder (BED) and OSFED as defined in the DSM-5 [15]. Of the 47 participants, 17 met the criteria for AN, 14 for BN, 3 for BED, and 13 for OSFED. Since there were only 3 participants with BED, BED and OSFED participants were united into one category. Diagnosis was first performed by a clinical psychologist and then by a research assistant using the EDE-Q. The AN group had a mean BMI of 16.75 (SD = 1.03), the BN group had a mean BMI of 22.74 (SD = 3.39), and the OSFED group had a mean BMI of 23.89 (SD = 4.57). Participants with EDs were between 18 and 47 years old.

The community sample consisted of 89 non-clinical females recruited through the use of social media platforms and the first year psychology student pool at Western Sydney University. The age range of this sample was between 18 and 45 years old and none had symptoms indicating a potential eating disorder.

### Measures

Participants were requested to complete the Schema Mode Inventory (SMI) and the Eating Disorder Examination Questionnaire (EDE-Q).

### Schema mode inventory

The SMI [16] is a self-report questionnaire that assesses the presence of 14 schema modes experienced by participants. For this sample, the SMI demonstrated an internal validity of α = .79 for the ED group, and α = .82 for the community sample.

### Eating disorder examination – questionnaire

EDE-Q [17] is a self-report questionnaire assessing four subscales: restraint, eating concern, shape concern, weight concern, and a global EDE-Q score, in addition to diagnostic measures of binge eating and purging. For this sample, the global EDE-Q demonstrated α = .95 for the ED group, and α = .93 for the community sample.

### Statistical analysis

Fourteen *t*-tests were used to compare SMI schema mode scores of the ED group with those of the community sample. A further five *t*-tests were used to compare the EDE-Q scores of the ED group and the community sample. To examine the effect size, Cohen’s d was calculated. Significance level was set to .05.

Additionally, planned contrasts within a series of one-way analysis of variance (ANOVA) were used to compare the fourteen SMI schema mode scores for the three types of diagnosis group and the community sample group. Bonferroni adjustments of .0167 were performed (*p* value of 0.05 was divided by 3 as there were 3 comparisons) to control for multiple comparisons.

## Results

### ED group and community sample on EDE-Q and SMI

The ED group scored significantly higher than the community sample on all four indexes of the EDE-Q, and the EDE-Q Global score. Additional *t*-tests showed that the ED group scored significantly higher than the community sample on 10 out of 12 (83 %) SMI schema modes classified as *maladaptive*, and significantly lower than the community sample on both of the SMI schema modes classified as *adaptive*. Means, standard deviations, *t*-values, and *p*-values obtained are presented in Table 1. Notably, the ED group showed no significant difference compared to the community sample on modes ‘Self-Aggrandiser’ and ‘Bully and Attack’. Large effect sizes were observed for modes ‘Vulnerable Child’, ‘Compliant Surrender’, ‘Detached Protector’, Detached Soother’, ‘Punitive Parent’, ‘Demanding Parent’ and ‘Healthy Adult’.

**Table 1:** Means, Standard Deviation and results for the comparisons between AN, BN, OSFED, and Community Sample groups on SMI and EDE-Q, and all EDs compared to the Community group

	Community sample	ED group		AN		BN		OSFED	
	*n* = 89	*n* = 47		*n* = 17		*n* = 14		*n* = 16	
	Mean (SD)	Mean (SD)	*t*-value	Mean (SD)	*t*-value	Mean (SD)	*t*-value	Mean (SD)	*t*-value
SMI									
Vulnerable Child	2.15 (0.73)	3.91 (1.16)	10.944**	4.02 (1.15)	8.086***	4.17(1.05)	8.012***	3.78 (1.16)	6.837***
Angry Child	2.29 (0.83)	2.91 (0.91)	4.569***	2.98 (1.22)	2.255	3.11 (0.84)	3.459**	2.73 (0.62)	2.582
Enraged Child	1.41 (0.48)	1.80 (0.86)	3.436**	2.04 (1.20)	2.135	1.70 (0.59)	1.717	1.69 (0.66)	1.606
Impulsive Child	2.30 (0.68)	2.70 (1.02)	2.777*	2.64 (1.15)	1.202	3.28 (0.96)	3.678**	2.28 (0.76)	-.086
Undisciplined Child	2.64 (0.83)	3.17 (0.88)	3.868***	3.24 (1.03)	2.885*	3.33 (0.84)	3.023**	3.02 (0.77)	1.759
Happy Child	4.25 (0.70)	2.78 (0.93)	−10.420***	2.46 (0.87)	−8.958***	2.69 (0.84)	−7.190***	3.02 (0.89)	−6.008***
Compliant Surrender	2.86 (0.66)	3.77 (0.97)	6.478***	4.05 (1.13)	4.177**	3.50 (0.75)	2.992*	3.83 (0.93)	3.967**
Detached Protector	1.93 (0.64)	3.46 (1.04)	10.602***	3.64 (1.06)	6.430***	3.67 (0.93)	6.724***	3.20 (1.02)	4.815***
Detached Soother	2.69 (1.01)	4.41 (0.92)	10.317***	4.28 (1.00)	6.097***	4.40 (0.99)	6.012***	4.53 (0.86)	6.882***
Self-Aggrandiser	2.65 (0.65)	2.79 (0.77)	1.190	2.76 (0.95)	0.461	2.90 (0.52)	1.635	2.73 (0.81)	0.384
Bully and Attack	1.98 (0.51)	1.95 (0.62)	−.321	1.91 (0.69)	-.490	2.20 (0.69)	1.348	1.77 (0.46)	−1.408
Punitive Parent	1.91 (0.62)	3.92 (1.14)	13.417***	4.09 (1.19)	7.382***	3.80 (1.00)	6.874***	4.01 (1.23)	6.637***
Demanding Parent	3.60 (0.82)	4.45 (0.86)	5.978***	4.56 (0.60)	4.340***	4.28 (0.73)	2.837*	4.44 (1.20)	3.699***
Healthy Adult	4.45 (0.65)	3.46 (0.82)	−7.715***	3.19 (0.87)	−6.828***	3.50 (0.70)	−4.728***	3.54 (0.74)	−4.830***
EDE-Q									
Dietary Restraint	2.79 (1.61)	3.71 (1.72)	3.097**						
Eating Concern	1.87 (1.02)	3.73 (1.65)	8.094***						
Shape Concern	3.43 (1.63)	4.74 (1.39)	4.646***						
Weight Concern	3.09 (1.57)	4.55 (1.55)	5.176***						
EDE-Q Global	2.80 (1.28)	4.18 (1.41)	5.779***						

*Note*: *ED* eating disorder, *AN* anorexia nervosa, *BN* bulimia nervosa, *OSFED* other specified feeding or eating disorder

* *p* < .05. ** *p* < .01. *** *p* < .001

The results of all planned contrasts are presented in Table 1. All Cohen’s d that reached significance ranged 0.55-2.24.

## Discussion

This study aimed to examine whether EDs have higher schema modes compared to a community sample, and to explore the relations between AN, BN, OSFED, and individual schema modes, each compared to a community sample.

As hypothesised, the results indicated that individuals with EDs scored significantly higher on 10 out of the 12 maladaptive modes compared to a community sample. Modes ‘Self-Aggrandiser’ and ‘Bully and Attack’, which centre on feeling superior and intimidating others, respectively [18], were not significantly different between groups, suggesting the possibility that this group of modes represent less-prominent mechanisms in the pathology of EDs. Perhaps superiority and intimidation of others are atypical of ED sufferers, thus rendering these modes less relevant to the ED group. Further, the ED group scored significantly lower on both schema modes identified as adaptive, including modes ‘Happy Child’ and ‘Healthy Adult’. Outcomes are comparable to that of Voderholzer et al. [14].

Similar patterns of modes were found for each ED, with AN, BN, and OSFED groups proving to be near identical in their modal associations. The BN group displayed a unique significant difference from the community sample on schema modes ‘Angry Child’ and ‘Impulsive Child’. This association was remarkably absent in both the AN and OSFED groups. Results suggest that impulsiveness, loss of control, and anger may characterise BN, but not AN or OSFED, signifying a unique schema mode profile for BN. These results are comparable to previous research that suggests an association between BN, anger and impulsivity [19, 20], and previous findings that give evidence for higher impulsivity scores amongst BN patients compared to AN patients [21]. The lack of association between the AN group and ‘Angry Child’ was unexpected as prior research has demonstrated a link between anger and AN [22]. The OSFED group displayed an additional absence of association with ‘Undisciplined Child’.

These numerous associations, combined with early success in clinical trials [13, 23, 24], highlight the notion that a mode-focused approach to schema therapy could be beneficial to individuals with EDs. Findings could also be used to further develop a mode-focused model of eating pathology that could potentially be akin to the mode-focused model of personality disorder proposed by Young [9, 24].

Limitations of this study include the use of a small sample size and the lack of a BED group. There are also limitations relating to the SMI in that it was designed for personality disorders, which may not highlight some of the coping modes present in ED patients. We are in the process of developing a schema mode inventory for EDs.

## Conclusions

Despite some limitations the current study showed a tendency for females with EDs to rely on maladaptive schema modes more frequently, and adaptive schema modes less frequently compared to a community sample. When comparing particular EDs (AN, BN, and OSFED groups) to a community sample, results were generally comparable. These conclusions provide preliminary support for mode-focused schema therapy in the treatment of individuals with EDs.

## Abbreviations

AN: Anorexia nervosa
BN: Bulimia nervosa
ED: Eating disorder
EDE-Q: Eating disorder examination questionnaire
OSFED: Otherwise specified feeding or eating disorder
SMI: Schema mode inventory
